# Implementation of a patient-focused psychosocial intervention guideline for people with severe mental illness: Cluster-randomised controlled trial

**DOI:** 10.1192/j.eurpsy.2025.10126

**Published:** 2025-10-23

**Authors:** Markus Kösters, Andreas Allgöwer, Thomas Becker, Reinhold Kilian, Uta Gühne, Steffi Riedel-Heller, Alkomiet Hasan, Peter Falkai, Klemens Ajayi, Peter Brieger, Karel Frasch, Theresa Halms, Stephan Heres, Markus Jäger, Andreas Küthmann, Albert Putzhammer, Bertram Schneeweiß, Michael Schwarz, Johanna Breilmann

**Affiliations:** 1 Center for Evidence-Based Healthcare, University Hospital Carl Gustav Carus, TU Dresden, Dresden, Germany; 2 Department of Psychiatry ll, Ulm University, Guenzburg, Germany; 3 Institute for Epidemiology and Medical Biometry, Ulm University, Ulm, Germany; 4 Department of Psychiatry, University of Leipzig Medical Center, Leipzig, Germany; 5 Institute of Social Medicine, Occupational Health and Public Health, Medical Faculty, University of Leipzig, Leipzig, Germany; 6 Department of Psychiatry, Psychotherapy and Psychosomatics, Faculty of Medicine, University of Augsburg, Augsburg, Germany; 7 DZPG (German Center for Mental Health), Partner Site München/Augsburg, Augsburg, Germany; 8 Department of Psychiatry and Psychotherapy, University Hospital Munich, Munich, Germany; 9 Kbo-Isar-Amper Hospital, Munich, Taufkirchen, Germany; 10 District Hospital Donauwörth, Donauwörth, Germany; 11 District Hospital Kempten, Kempten, Germany; 12 District Hospital Memmingen, Memmingen, Germany; 13 Department of Psychiatry, Psychotherapy and Psychosomatics, District Hospital Kaufbeuren, Kaufbeuren, Germany

**Keywords:** empowerment, implementation, patient version of a clinical guideline, Psychosocial interventions, severe mental disorders

## Abstract

**Background:**

Psychosocial interventions are vital in treating severe mental illness, yet their use remains limited, and patients often lack adequate information about them. Patient-focused versions of clinical guidelines are designed to enhance mental health literacy and inform patients about available treatments, but these resources are underutilized. This study evaluated the impact of implementing a patient-focused psychosocial intervention guideline on empowerment, knowledge, and use of psychosocial interventions among individuals with severe mental illness.

**Methods:**

Multicentre, cluster-randomised trial. The study population comprised adult patients with a severe mental disorder. The intervention group received a multimodal, structured, and protocol-led patient-focused guideline implementation, whereas the control group received treatment as usual. Data were analysed using hierarchical linear models. The primary outcome was the change in patients’ empowerment.

**Results:**

There was no significant intervention effect on empowerment (effect size=0.13, p=0.605), which increased slightly in both groups. The number of psychosocial interventions familiar to patients increased significantly more in the intervention group. Exploratory analyses suggest that patient empowerment could have been influenced by COVID-19-related stress, patient age, the severity of functional impairment, and migration background. The improvement in the utilisation of psychosocial interventions did not differ significantly between the intervention group (M=1.1, SD=2.5) and the control group (M=1.3, SD=2.4).

**Conclusions:**

The implementation of a patient-focused psychosocial intervention guideline failed to enhance empowerment among service users. However, our analyses indicate that the intervention led to an improvement in patient knowledge with respect to guideline content. The availability of psychosocial interventions may have been significantly constrained by the COVID-19 pandemic.

## Introduction

Psychosocial interventions are essential for treating severe mental illnesses, enhancing individuals’ social functioning and societal participation [1]. These interventions include a variety of interventions, such as occupational therapy, art therapy, multidisciplinary team-based community mental health interventions, and supported employment [1]. In 2012, the first edition of the German evidence- and consensus-based guideline “Psychosocial interventions in severe mental disorders” was published, along with a patient version of the guideline in plain language [2]. Both versions have been updated in 2019 [3, 4]. However, evidence suggests that utilisation rates of psychosocial interventions [5] are low, and patients in Germany are insufficiently informed about these interventions [6]. Fritz et al. (2025) found that for four out of nine interventions studied, below 25% of patients received the interventions. For two interventions, approximately 50% of patients received these, and for only three interventions, the majority (75–90%) of patients received these interventions [5]. Improved knowledge of treatment options is crucial to enhance mental health literacy, service utilisation, and shared decision-making [7]. Accompanying self-help and self-care are vital for patients’ empowerment and recovery [7]. Patient-focused guidelines are one tool to improve patients’ mental health literacy and knowledge of existing treatment options [8], but a lack of awareness among patients poses a significant barrier to their use [7].

While the pathway of guideline development has become highly sophisticated, evidence on their clinical implementation, particularly in mental health care, is sparse and inconsistent [9]. There is also a lack of evidence on the implementation of patient-focused guideline versions. To date, no controlled study has examined the influence of structured patient-focused guideline implementation on empowerment (perceived self-determination), treatment utilisation, or other outcomes in psychiatry. However, some studies have shown encouraging positive effects of patient information or patient brochures in guideline implementation [10].

This cluster-randomised controlled trial aimed to evaluate the effects of a multimodal, structured patient-focused guideline implementation (intervention) versus treatment as usual (control condition) on the empowerment among patients with severe mental illness (primary outcome) in 10 clinics (clusters). Empowerment is a core component of recovery [11], and access to quality information on treatment options [12] is crucial for patient empowerment. Therefore, we hypothesised that the patient-focused guideline implementation would enhance empowerment in the intervention group. Given the small effects in prior studies using empowerment as a primary outcome [13], we also assessed differences in effects on patient knowledge of treatment options and the utilisation of psychosocial interventions (secondary outcomes). A cluster-randomised design at the recruiting centre (clinics) level was used to prevent spill-over effects between intervention and control groups.

## Methods

The reporting of the IMPPETUS study (“Implementation of the patient guideline psychosocial interventions for patients with severe mental illness”) followed the CONSORT guideline extended for cluster-randomised trials [14] (see Supplementary Table S5). The study and protocol were registered before recruitment in the International Clinical Trials Registry Platform (ICTRP) (No. DRKS00017577; https://trialsearch.who.int/Trial2.aspx?TrialID=DRKS00017577) and published [15].

### Study design and setting

This multicentre, cluster-randomised, controlled, open-label, two parallel-groups-superiority trial collected data from 10 psychiatric clinics in Upper Bavaria and Swabia in Germany, covering metropolitan (Augsburg, Munich), mid-urban (Kempten, Memmingen), and rural areas (Donauwoerth, Guenzburg, Kaufbeuren, Taufkirchen).

### Randomisation

The Institute of Epidemiology and Medical Biometry at Ulm University conducted external randomisation of study centres (clinics) using stratified block randomisation, assigning the 10 clinics 1:1 to intervention or control groups based on clinic size (small, large) via the ROM [16] software.

### Eligibility criteria

We included inpatients and day hospital patients of the 10 clinics with severe mental disorder as defined by the guideline “Psychosocial interventions in severe mental disorders” [1] (disease duration of ≥2 years and significant impact on activities of daily life). However, we limited the inclusion criteria to patients with routine clinical diagnoses of schizophrenia, schizotypal and delusional disorders (ICD-10 F2x) or mood (affective) disorders (ICD-10 F3x) to ensure a more homogenous sample [17]. Significant impact on activities of daily living was defined as a “Global Assessment of Functioning” (GAF) [18] score ≤ 60 and a “Health of the Nation Outcome Scales” (HoNOS) [19] fulfilling one of two conditions: (a) a score of ≥2 on one of the items of the subscale for symptomatic problems (item 6, 7, and 8) and a score of ≥2 on each of the four items of the subscale for social problems (items 9, 10, 11, and 12); or (b) a score of ≥3 on at least one of these items (9, 10, 11, or 12). Patients of all genders aged 18–65 years were eligible, provided they could understand the research project and decide on participation. The responsible physician determined capacity to consent when in doubt, and legal representatives were informed if patients consented.

Relatives of all genders aged over 18 years, understanding the research project, and not currently in inpatient or day hospital mental health care were included to incorporate their perspective. However, this paper reports on patient results only.

### Recruitment procedure

Recruitment began post-randomisation on 28/10/2019, with the final follow-up in September 2021. Patients from the randomised clinics were invited to participate in the study and to provide consent for participation in the trial and follow-up 6 months after baseline assessment. Following written consent, study participants were screened by GAF and HoNOS to ensure they met the criteria of severe mental illness. We screened patients at the earliest possible time after admission.

### Intervention

The patient guideline “Psychosocial interventions in severe mental disorders” [2] was implemented using a structured, multimodal strategy in a clinic cluster-randomised to the experimental group. Participants received the intervention approximately 2–4 weeks after the baseline assessment (t0). An educational group intervention for patients was developed (including a detailed protocol), comprising two 60-minute sessions across five modules [20]. Due to altered conditions during the COVID-19 pandemic, sessions were adapted to be flexible, allowing the content to be covered in a single 90-minute session. The delivery of the sessions was continuously monitored and adapted. The goal was to present the guideline content in a patient-friendly manner, delivered jointly by study staff and a peer tutor. Modules 1–4 covered illness concepts, the various psychosocial interventions, guideline background, and personal resources and goals, using methods to engage participants. Module 5 addressed regional variations in psychosocial service availability, providing participants with information about all local services. Decision aids and reminders were created to support the application of knowledge in treatment discussions and encourage requests for psychosocial interventions that are oriented towards individual patient needs. These aid tools were practiced during group sessions with participants. Group sessions were offered regularly in inpatient and day hospital settings. Participants were encouraged to attend the educational group at least once in full. Participants also received a printed copy of the patient guideline [2] and the waiting room version of the guideline [21]. In addition, key content of the patient guideline was made available in the form of the online intervention tool “TheraPart” [22]. Additionally, a 1-hour information session was offered to relatives.

Participants in clinics assigned to the control condition received standard care, but were informed about the patient guideline “Psychosocial interventions in severe mental disorders” [2] using a flyer (waiting room version) [21], which was also offered to study participants in the intervention group. These flyers summarise the key information of the guideline and refer to additional information.

Study staff underwent comprehensive training for the implementation of study conditions to ensure consistency across sites, with fidelity monitored through regular re-training and supervision.

### Data collection and management

Patients were interviewed at three time points: (1) t0 = shortly after admission (baseline); (2) t1 = intervention group: 2 weeks after attending the group sessions; control group: 6 weeks after t0; and (3) t2 = 6 months after t0 (both groups). Data were initially recorded on paper, then entered into a standardised form at the recruitment centres and checked by a second team member to prevent errors. The data were securely stored in a computer database at the study centre.

To minimise loss to follow-up, study staff regularly contacted study participants, visiting them during their hospital stay and informing them post-discharge via newsletters, phone, or email about upcoming data collection. Post-discharge assessments could be done at the study participants’ homes or by telephone.

More details of the assessments at each time point are reported in the protocol [15].

### Outcomes and measurement

This paper examines changes in empowerment (primary outcome) and in patients’ knowledge and use of psychosocial interventions (secondary outcomes). All outcomes and demographics assessed are detailed in the study protocol [15].

The change in empowerment was measured on the “empowerment in the process of psychiatric treatment of patients with affective and schizophrenia disorders” (EPAS) scale [23]. The EPAS base module includes 33 items, covering five dimensions: “everyday coping,” “social relationships,” “treatment participation,” “self-esteem,” and “self-efficacy.” Two additional modules cover empowerment in caring for minor children and at work. The EPAS scale’s psychometric properties were validated in a field study [23].

Changes in patients’ knowledge and use of psychosocial interventions were assessed using a checklist developed for this study, asking, with respect to all psychosocial interventions, whether patients knew about them, had received them, and found the interventions they had received helpful.

The COVID-19 pandemic affected study recruitment. To estimate its impact, a short “corona burden index” (CBI) was developed, consisting of eight questions on the pandemic burden, rated on a 5-point scale from “no restriction”/“strongly disagree” to “strongly agree.” This questionnaire was used for interviews from May 2020 onwards. The value “no restriction” was used for pre-March 2020 interviews. The CBI was only included in the model as a covariate to assess whether the subjective burden of COVID-19 restrictions had an impact on the results, and was not used as an outcome.

The characteristics of the participants (gender, age, etc.) were recorded using the “Client Sociodemographic and Service Receipt Inventory” (CSSRI) [24]. Diagnoses were taken from the medical records.

### Sample size calculation

The sample size calculation used a two-step procedure due to the hierarchical data (cluster). Initially, a sample size for an individual randomised trial was calculated. Based on studies using the EPAS [13], a medium effect (0.4 SD units) of the implementation intervention on the primary endpoint was expected. This effect is detectable with an error probability of 5% (alpha) and a power of 80% by a t-test for independent samples with a sample size of 2x100 participants (200 in total).

In a second step, the cluster structure of the data was considered. We assumed that each centre (cluster) recruits 35 patients (m) on average. The design effect (DE) was calculated as DE = 1 + (m − 1) × ICC with the fixed cluster size m and an intra-cluster correlation coefficient (ICC) of 0.02, resulting in DE = 1.68. This gave a resulting sample size of 200 x 1.68 = 336 patients. Considering a 70% follow-up rate at 6 months after t0 (t2) due to the severity of the disease in the patient population and based on experience in previous studies, we aimed to recruit 50 patients per study centre, totalling 500 participants (2 × 250) across 10 centres. Clusters of equal size were assumed.

### Statistical methods

All statistical analyses were planned a priori [15]. Sociodemographic and clinical characteristics are listed, with categorical variables as absolute and relative frequencies, and continuous variables as means (M) and standard deviations (SD).

The primary outcome was the improvement in empowerment at 6 months after t0 (t2) (EPAS6) compared to baseline (t0) (EPAS0). A hierarchical linear model (mixed model approach [25,26]) was used, modelling EPAS6 as the outcome. Cohen’s d was used to calculate the effect size. The group (cluster) assignment (intervention group/control group), the baseline EPAS (“EPAS0”), and the size of the study centre region (“study centre size”: metropolitan, middle-urban, rural) were used as main regressors with special attention to the group assignment (confirmatory). The significance level was set at 5% for the group assignment. The analysis was based on a modified intention-to-treat (modITT)-population, using all patients with an outcome at month 6 (t2) (EPAS6) [27], as imputation of the primary outcome may be misleading. To assess for potential selection bias, the baseline characteristics of the ITT and modITT populations were compared. According to this modITT approach, multiple imputation [28] of missing data in independent variables was performed by fully conditional specification (FCS) in order to perform a sensitivity analysis. Due to a suggestion in the review process, we also conducted a sensitivity analysis with imputed outcome data. Other potential confounders of the primary outcome (e.g. CBI, age, GAF score, diagnosis [depression/ schizophrenia/ bipolar disorders], level of school education [ongoing or no graduation/low/medium/high], migration background [yes/no]) were examined in exploratory analyses by including them in the model, using backward selection with variable exclusion at p > 0.1 and multiple imputation where appropriate (see above).

The secondary outcomes were analysed accordingly.

## Results

### Study sample

For patient flow, see Supplementary Figure S1. Baseline data (t0) were collected for 534 patients (*n* = 260 in clinics of the intervention group; *n* = 274 in clinics of the control group), 405 patients (76% of 534) were assessed at t1 (post-intervention) (*n* = 192 in clinics of the intervention group; *n* = 213 in clinics of the control group), and 356 patients (67% of 534) were assessed at t2 (6-month assessment) (*n* = 178 in clinics of the intervention group; *n* = 178 in clinics of the control group).

In the intervention group, 208 of 260 (80%) patients took part in the intervention. Of the 178 intervention group patients with follow-up data at 6 months (t2), 163 patients (91.6%) received the full intervention content, eight patients (4.5%) received one of two sessions, and seven patients (3.9%) did not participate in the intervention (Supplementary Figure S1).

A total of 178 patients (33%) (*n* = 82 in clinics of the intervention group; *n* = 96 in clinics of the control group) withdrew from the study after baseline, because they could no longer be contacted or they actively refused to continue their study participation. These patients were significantly younger (*U* = 26215.5, *Z* = −3.160, *p* = 0.002) and more often had a migration background (*χ*^2^(1) = 6.551, *p* = 0.010) than the patients remaining in the study (Supplementary Table S1).

Data were available for 356 patients for the primary analysis. The characteristics of this sample are described in Table 1.

**Table 1.** Patient characteristics at baseline

	Intervention group (5 clinics)		Control group (5 clinics)	
	*n* ^a^	*n* (%) or M (SD)	*n* ^a^	*n* (%) or M (SD)
**Gender, *n* (%)**	178		178	
Men		67 (37.6)		79 (44.4)
Women		111 (62.4)		99 (55.6)
**Age [years], *M* (SD)**	178	42.2 (12.9)	178	43.6 (12.8)
**Diagnosis, *n* (%)**	178		178	
Depression		137 (77.0)		106 (59.6)
Schizophrenia		31 (17.4)		58 (32.6)
Bipolar disorders		10 (5.6)		14 (7.9)
**Psychosocial functioning [GAF-Scale], *M (SD)***	178	44.8 (7.6)	178	41.7 (8.8)
**Duration of illness [years], *M* (SD)**	178	15.4 (11.8)	178	17.9 (12.2)
**Level of school education** ^b^ **, *n* (%)**	178		178	
Ongoing or no graduation		2 (1.1)		8 (4.5)
Low		48 (27.0)		55 (30.9)
Medium		70 (39.3)		48 (27.0)
High		58 (32.6)		67 (37.6)
**Migration background, *n*** (%)	178		177	
Yes		26 (14.6)		30 (16.9)
No		152 (85.4)		147 (83.1)
EPAS^**c**^ **, *M (SD)***	178	3.3 (0.6)	178	3.3 (0.6)
**Familiar with the term “psychosocial interventions?” *n* (%)**	178		178	
Yes		83 (46.6)		93 (52.2)
No		95 (53.4)		85 (47.8)

Abbreviations: M = Mean, SD = standard deviation.

a *n* patients.

b According to the German school system: ongoing or no graduation = still in school or no school leaving certificate; Low = graduation after 9 years of education; Medium = graduation after 10 years of education; High = high school graduation.

c Mean of the base module with 33 items only, without the two additional modules (empowerment in caring for minor children and at work).

### Primary outcome – empowerment

The analyses show that patient empowerment (EPAS total score) increased minimally in both groups. The mean total EPAS score increased by 0.28 points (95% Confidence interval [CI]: 0.20–0. 37) in the control group and by 0.35 points (95% CI: 0.27–0.73) in the intervention group (effect size = 0.13; Supplementary Table S2). There was no statistically significant difference in improvement between the groups (*p* = 0.605; Table 2). The sensitivity analyses showed no meaningful differences compared to the modITT population.

**Table 2.** Analyses of the primary outcome “empowerment” (EPAS)

		Hierarchical linear models^a^		Exploratory backward selection procedure^b^	
		*Estimate (SE)* ^a^	*p-value*	*Estimate (SE)*	*p-value*
***Fixed Effects***					
**Intercept**		1.506 (0.152)	< 0.001	1.603 (0.239)	< 0.001
**Group assignment**	***Intervention (vs. control***)	−0.045 (0.088)	0.605		
**EPAS0^c^**		0.634 (0.042)	**< 0.001**	0.642 (0.042)	**< 0.001**
**Study centre size**	***Metropolitan (vs. rural)***	−0.033 (0.085)	0.697		
	***Middle-urban (vs. rural)***	0.211 (0.118)	0.074		
**CBI2^d^**				−0.052 (0.029)	0.073
**Age**				−0.006 (0.002)	**0.006**
**GAF**				0.008 (0.003)	**0.023**
**Migration background**	***Yes (vs. no)***			−0.158 (0.070)	**0.025**
***Error variance***					
**Level 1**		0.230 (0.018)	< 0.001	0.205 (0.016)	< 0.001
**Level 2 intercept**		0.006 (0.008)	0.205	0.006 (0.007)	0.169

*Note:* ICC = 0.046.

Abbreviations: SE = Standard error, bold: statistically significant influence (p<0.05).

a For the confirmatory main analysis; *n* = 353 patients (10 clinics).

b *n* = 328 patients (10 clinics).

c EPAS0 = EPAS score at baseline (t0).

d CBI2 = “corona burden index” at t2.

In exploratory analyses, the EPAS score at baseline (*p* < 0.001), age (*p* = 0.006), disability level (GAF) of patients (*p* = 0.023), and migration background (*p* = 0.025) showed a significant influence on the change in EPAS total score (Table 2). A higher baseline EPAS score and a higher level of patient functioning (GAF score) are associated with an increase in empowerment. Older patients and patients with a migration background showed a decrease in empowerment. The CBI at t2 showed a tendency to decrease empowerment (*p* = 0.073).

### Knowledge of psychosocial interventions

After 6 months (t2), the term “psychosocial intervention” was known to more patients in the intervention group than in the control group (84% versus 75%). Across all psychosocial interventions, the number of psychosocial interventions familiar to study participants improved more in the intervention group than in the control group (mean improvement 6.5 (SD 4.8) psychosocial interventions known versus 2.9 (SD 4.1) (Supplementary Table S3). This was also seen in the hierarchical linear model (Table 3; *p* = 0.013). An increase in knowledge was also seen for those psychosocial interventions that were less well known at baseline, such as “lifestyle interventions” or “treatment by multidisciplinary community mental health teams.” Also, for individual types of psychosocial interventions, there was an increase in knowledge in both study groups, but in most cases the increase was larger in the intervention group (e.g. “support for self-management” from 27 to 75% versus 38 to 63%). In the descriptive analyses, interventions well known at baseline, for example, “self-help groups” or “art therapy,” were the only ones that failed to show differential benefits regarding patient knowledge (in the intervention group).

**Table 3.** Analyses of the secondary outcome “knowledge”

		Hierarchical linear models^a^		Backward selection procedure^b^	
		*Estimate (SE)*	*p-value*	*Estimate (SE)*	*p-value*
***Fixed effects***					
**Intercept**		2.572 (0.886)	0.027	−2.247 (2.045)	0.304
**Group assignment**	***Intervention (vs. control)***	3.065 (1.223)	**0.013**	3.205 (0.837)	**< 0.001**
**Study centre size**	***Metropolitan (vs. rural)***	0.681 (1.186)	0.567		
	***Middle-urban (vs. rural)***	1.817 (1.659)	0.274		
**Age**				−0.039 (0.019)	**0.038**
**Diagnosis**	***Depression (vs. schizophrenia)***			1.837 (0.578)	**0.002**
	***Bipolar disorders (vs. schizophrenia)***			0.468 (0.995)	0.639
**Level of school education^c^**	***High (vs. ongoing or no graduation)***			3.623 (1.424)	**0.011**
	***Medium (vs. ongoing or no graduation)***			2.900 (1.433)	**0.044**
	***Low (vs. ongoing or no graduation)***			3.231 (1.442)	**0.026**
**GAF**				0.059 (0.033)	0.071
***Error variance***					
**Level 1**		18.979 (1.457)	< 0.001	17.983 (1.399)	<.0001
**Level 2 intercept**		1.990 (1.487)	0.090	1.114 (0.905)	0.1091

*Note:* ICC = 0.222.

Abbreviation: SE = Standard error, bold: statistically significant influence (p<0.05) .

a For the exploratory main analysis; *n* = 349 patients (10 clinics).

b *n* = 348 patients (10 clinics).

c According to the German school system: ongoing or no graduation = still in school or no school leaving certificate; Low = graduation after 9 years of education; Medium = graduation after 10 years of education; High = high school graduation.

In exploratory analyses, group assignment (*p* < 0.001), age (*p* = 0.038), a diagnosis of depression versus schizophrenia (*p* = 0.002), and completed school education versus ongoing or no graduation (high education: *p* = 0.011; medium education: *p* = 0.044, low education: *p* = 0.026) were shown to influence the improvement in knowledge on psychosocial interventions (Table 3).

### Utilisation of psychosocial interventions

Patients’ reported participation in psychosocial interventions shows an inconsistent picture. Only for a small number of psychosocial interventions, which a few patients received at baseline (e.g. “self-management support”), utilisation increased slightly, with no difference between study groups. Across all psychosocial interventions, there was no meaningful difference with respect to increased participation in psychosocial interventions across groups (mean improvement 1.1 (SD 2.5) for the intervention group versus 1.3 (SD 2.4) for the control group; Supplementary Table S4), which was also seen in the analysis (Table 4; *p* = 0.746).

**Table 4.** Analyses of the secondary outcome “utilisation”

		Hierarchical linear models^a^		Backward selection procedure^b^	
		*Estimate (SE)*	*p-value*	*Estimate (SE)*	*p-value*
***Fixed effects***					
**Intercept**		1.555 (0.485)	0.024	0.775 (0.336)	0.050
**Treatment**	***Intervention (vs. control)***	0.198 (0.611)	0.746		
**Study centre size**	***Metropolitan (vs. rural)***	−0.467 (0.606)	0.441		
	***Middle-urban (vs. rural)***	−1.163 (0.824)	0.159		
**Diagnosis**	***Depression (vs. schizophrenia)***			0.631 (0.310)	**0.042**
	***Bipolar disorders (vs. schizophrenia)***			−0.051 (0.559)	0.928
***Error variance***					
**Level 1**		5.645 (0.445)	< 0.001	5.591 (0.442)	< 0.001
**Level 2 intercept**		0.448 (0.383)	0.121	0.391 (0.271)	0.075

Note: ICC = 0.065.

Abbreviation: SE = Standard error, bold: statistically significant influence (p<0.05).

a For the exploratory main analysis; *n* = 331 patients (10 clinics).

b *n* = 331 patients (10 clinics).

In exploratory analyses, only the diagnosis of depression versus schizophrenia showed a significant influence on the improvement of participation in psychosocial interventions (*p* = 0.042; Table 4).

## Discussion

The intervention designed, in this trial, to implement the patient-focused version of a national evidence- and consensus-based psychosocial intervention guideline [2] failed to significantly improve empowerment among service users in the intervention group over and above the control group. Exploratory analyses indicate that patients’ empowerment may have been influenced by COVID-19-related stress, patient age, the severity of functional impairment, and migration background. However, the intervention did improve knowledge of guideline content, although actual uptake of psychosocial interventions varied. The availability of these interventions may have been restricted by the COVID-19 pandemic. The high level of empowerment among participants in both groups may have led to a ceiling effect.

Medical treatment guidelines summarise the evidence that is currently available and formulate evidence- and consensus-based recommendations on the basis of an aggregate reading of the evidence. The idea is that evidence-based guidelines should guide practitioners of a given field of medicine in their information and treatment recommendations offered to people experiencing conditions of ill health. The majority of guideline implementation trials have sought to use the guideline versions formulated for clinicians, while only a few implementation trials have used the patient versions of guidelines and thus targeted service users (or wider medical care provider organisations). The present trial used the patient-focused version of a national psychosocial intervention guideline targeting the group of people with severe mental illness [2]. The guideline implementation intervention used a one- to two-session joint peer plus study worker intervention supplemented with TheraPart [22], an online intervention tool that was delivered to all study participants and that was, in fact, increasingly being accessed in the course of the study. Intervention uptake and fidelity to the intervention protocol were high, and the complete intervention was received by 91.6% of study participants in the intervention group.

Knowledge is considered by many to be an important prerequisite of adequate health behaviour and a key component of agency in the field of health care in a population [7]. The differential improvement with respect to knowledge (about psychosocial interventions) in the intervention group suggests that the intervention “did something,” that some of the content was not forgotten, and that study participants, in the intervention group, were more likely to have that resource in hand after participation in the IMPPETUS trial. However, it seems that improving knowledge, although a core component of empowerment, is not sufficient to also affect self-reported empowerment.

Community mental health care relies on a range of complex psychosocial interventions delivered by multidisciplinary teams. This applies to team-based community care, both long-term and in acute crisis, as well as to supported housing services, supported education, supported employment, and integrated peer support interventions. Patients’ access to these interventions depends significantly on the mental health care system, its organisation, the peculiar monocultural or mixed economy of care configuration of the service, the specific nature and degree of integration of mental health and social service systems, the purchaser or finance regulations and the statutory, national, regional, or local rules governing service provision, uptake, and funding. Treatment guidelines can only go some of the way in determining which interventions service users will be able to access in a given situation of ill health. This is true for the full range of treatment options (pharmacological treatment, psychotherapy), and it is clearly true for psychosocial interventions. Guidelines can, however, promote the improvement of evidence-based health services, but such changes only slowly prevail against structures that have grown over many years.

The German mental health care system does not routinely rely on the full range of team-based community and psychosocial interventions. In the cross-sectional study to prepare this RCT, there was an observation that interventions supported by the strongest aggregate scientific evidence and by the strongest recommendations supporting their use were at the lower end with respect to the proportion of study participants receiving them across study sites [5]. Thus, service uptake was counter-intuitive with respect to the recommendations of the very guideline that was to be implemented in the IMPPETUS trial.

### Limitations

The study’s results must be viewed considering methodological limitations. First, although clinical data (e.g. GAF values) indicated severe illness, selection bias may be present as only patients who felt well enough to participate may have done so. Moreover, the sample included only patients who were (fully or day-) hospitalised, limiting generalisability to all patients with severe mental disorders. Patients with personality, anxiety, and obsessive-compulsive disorders were excluded, despite being part of the guideline’s target group. Additionally, participants needed proficiency in German. It should also be noted that all recruitment clinics were located in Bavaria, which may further limit the generalisability of the results to the entire group of severely mentally ill people in Germany. Furthermore, the multiple interviews conducted (baseline and follow-up) might have altered response behaviour, which could be described as a test effect. In addition, the study effect may have been weakened by distributing the patient guideline flyer to the participants in the control group. Lastly, the extent to which the COVID-19 pandemic influenced the results remains unclear. While perceived burden was included to estimate the pandemic’s impact, the full extent may not have been captured.

## Conclusion

By increasing patients’ knowledge, our study showed that it is feasible to directly target severely mentally ill patients within a structured implementation strategy. However, the intervention did not lead to a significant increase in empowerment among service users in the intervention group, suggesting that improving knowledge is not sufficient to increase empowerment and service use.

The findings of the present study do, in fact, caution against placing undue emphasis on (and reliance on) treatment guideline recommendations in the attempt to enhance the utilisation of psychosocial interventions among people with severe mental illness in Germany (or in mental health care systems with characteristics similar to the German one). The German care system is characterised by a strong focus on inpatient care and may therefore not be sufficiently flexible, which makes it difficult for patients to help shape their use of psychosocial interventions. However, there is hope now as services can reach higher levels of flexibility in budgetary use [29] (given a defined number of patients are seen by the comprehensive mental health service and cared for with adequate standards). Under such conditions, mental health care practice can be shifted from mostly inpatient care to higher levels of community-based service provision. This type of enhanced flexibility is likely to increase the availability of team-based psychosocial care in a given catchment area and, thus, to increase the freedom in clinical teams to freely use community-based psychosocial interventions when deemed adequate in the future.

## Supporting information

10.1192/j.eurpsy.2025.10126.sm001 Kösters et al. supplementary material Kösters et al. supplementary material

## Data Availability

The data that support the findings of this study are available on request from the corresponding author, M.K. The data are not publicly available due to participant privacy concerns.

## Supplementary material

The supplementary material for this article can be found at http://doi.org/10.1192/j.eurpsy.2025.10126.
