# Supplementary material for: Implementation of a patient-focused psychosocial intervention guideline for people with severe mental illness: Cluster-randomised controlled trial
Source: Eur Psychiatry. 2025 Oct 23;68(1):e163. doi: 10.1192/j.eurpsy.2025.10126 (PMC12646118; doi:10.1192/j.eurpsy.2025.10126)
Supplement: Kösters et al. supplementary material [file S0924933825101260sup001.pdf]

Supplementary Material

Koesters et al.

Implementing the patient version of the guideline for psychosocial interventions for patients with severe mental illness - results from a cluster randomised controlled trial

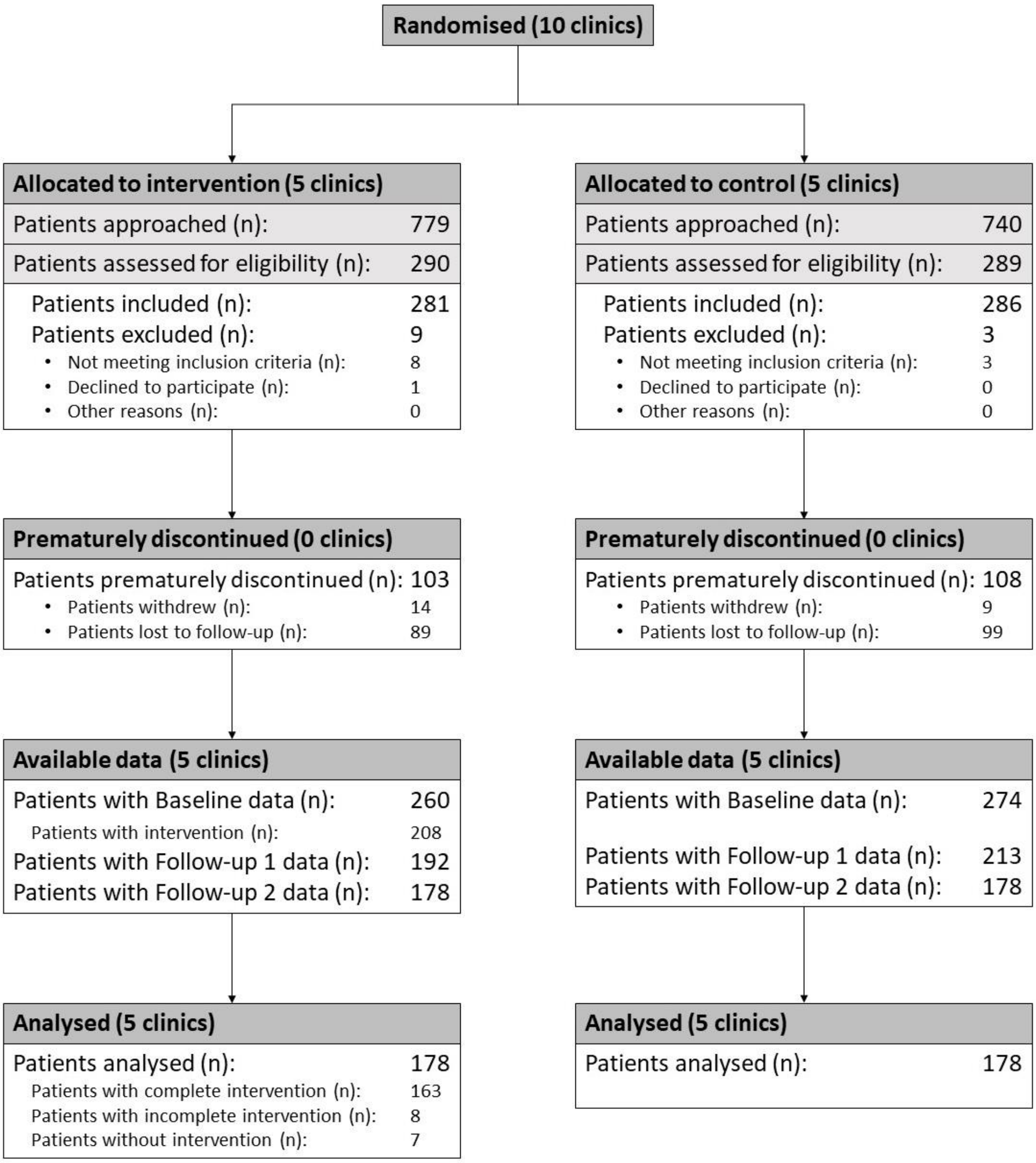

Supplementary Fig S1: Patient flow chart

Supplementary Table S1: Characteristics of dropouts in comparison to non-dropouts (baseline data). n = 10 clinics

	**Non-Dropouts**		**Dropouts**		
	***n*[1]**	***n (%) or M (SD)***	***n*[1]**	***n (%) or M (SD)***	***p-value***
**Sex, *n (%)***	356		177		
Men		146 (41.0)		80 (45.2)	
Women		210 (59.0)		97 (54.8)	0.357[2]
**Age [years], *M (SD)***	356	42.9 (12.9)	178	39.1 (13.2)	**0.002**[3]
**Diagnosis, *n (%)***	356		178		
Depression		243 (68.3)		113 (63.5)	
Schizophrenia		89 (25.0)		51 (28.7)	
Bipolar Disorders		24 (6.7)		14 (7.9)	0.543[2]
**Psychosocial functioning [GAF-Scale], *M (SD)***	356	43.3 (8.4)	178	42.1 (8.1)	0.109[3]
**Duration of illness [years], *M (SD)***	356	16.7 (12.1)	178	15.7 (11.1)	0.469[3]
**Level of school education[4], *n (%)***	356		176		
ongoing or no graduation		10 (2.8)		8 (4.5)	
low		103 (28.9)		56 (31.8)	
medium		118 (33.1)		59 (33.5)	
high		125 (35.1)		53 (30.1)	0.520[2]
**Migrant background, *n (%)***	355		176		
Yes		56 (15.8)		44 (25.0)	
No		299 (84.2)		132 (75.0)	**0.010**[2]
**EPAS[5], *M (SD)***	356	3.3 (0.6)	178	3.3 (0.7)	0.584[3]
**Familiar with the term "psychosocial interventions"? *n (%)***	356		170		
yes		176 (49.4)		75 (44.1)	
no		180 (50.6)		95 (55.9)	0.253[2]

M = Mean, SD = standard deviation

[1] n patients

[2] Pearson's chi-quadrat test

[3] Mann-Whitney U Test

[4] According to the German school system: ongoing or no graduation = still in school or no school leaving certificate; Low = graduation after 9 years education; Medium = graduation after 10 years education; High = high school graduation

[5] Mean of the base module with 33 items only, without the two additional modules (empowerment in caring for minor children and at work).

Supplementary Table S2: Group size and mean (standard deviation) of *empowerment* (as measured by the EPAS) at baseline (t0), six months after baseline (t2), and their difference. n = 10 clinics

	***n*[1]**	**Baseline (t0)**	***n*[1]**	**Six months after baseline (t2)**	***n*[1]**	**Difference**
**Overall**	353	3.29 (0.62)	354	3.61 (0.63)	353	0.32 (0.54)
**Intervention group**	178	3.26 (0.61)	178	3.61 (0.64)	178	0.35 (0.51)
**Control group**	175	3.31 (0.63)	176	3.60 (0.62)	175	0.28 (0.60)

[1] n patients

Supplementary Table S3: Group size and mean (standard deviation) of *knowledge* of psychosocial interventions at baseline (t0), six months after baseline (t2), and their difference. n = 10 clinics

	***n*[1]**	**Baseline (t0)**	***n*[1]**	**Six months after baseline (t2)**	***n*[1]**	**Difference**
**Knowledge**						
**Overall**	349	14.24 (4.31)	351	19.00 (4.84)	349	4.74 (4.83)
**Intervention group**	176	13.66 (4.22)	177	20.18 (4.48)	176	6.52 (4.81)
**Control group**	173	14.83 (4.34)	174	17.80 (4.91)	173	2.93 (4.13)

[1] n patients

Supplementary Table S4: Group size and mean (standard deviation) of *utilisation* of psychosocial interventions at baseline (t0), six months after baseline (t2), and their difference. n = 10 clinics

	***n*[1]**	**Baseline (t0)**	***n*[1]**	**Six months after baseline (t2)**	***n*[1]**	**Difference**
**Overall**	331	7.31 (3.30)	337	8.48 (3.07)	331	1.20 (2.45)
**Intervention group**	171	7.32 (3.04)	175	8.38 (2.72)	171	1.09 (2.50)
**Control group**	160	7.31 (3.58)	162	8.59 (3.42)	160	1.31 (2.39)

[1] n patients

Supplementary Table S5: CONSORT 2010 checklist of information to include when reporting a cluster randomised trial

Section/topic and item No	Standard checklist item	Extension for cluster designs	Page No*
**Title and abstract**			
1a	Identification as a randomised trial in the title	Identification as a cluster randomised trial in the title	1
1b	Structured summary of trial design, methods, results, and conclusions (for specific guidance see CONSORT for abstracts)[11 12]	See table 2	2
**Introduction**			
Background and objectives:			
2a	Scientific background and explanation of rationale	Rationale for using a cluster design	3
2b	Specific objectives or hypotheses	Whether objectives pertain to the cluster level, the individual participant level, or both	3
**Methods**			
Trial design:			
3a	Description of trial design (such as parallel, factorial) including allocation ratio	Definition of cluster and description of how the design features apply to the clusters	4
3b	Important changes to methods after trial commencement (such as eligibility criteria), with reasons		N/A
Participants:			
4a	Eligibility criteria for participants	Eligibility criteria for clusters	4, 5
4b	Settings and locations where the data were collected		4
Interventions:			
5	The interventions for each group with sufficient details to allow replication, including how and when they were actually administered	Whether interventions pertain to the cluster level, the individual participant level, or both	5/6
Outcomes:			
6a	Completely defined prespecified primary and secondary outcome measures, including how and when they were assessed	Whether outcome measures pertain to the cluster level, the individual participant level, or both	7
6b	Any changes to trial outcomes after the trial commenced, with reasons		7
Sample size:			
7a	How sample size was determined	Method of calculation, number of clusters(s) (and whether equal or unequal cluster sizes are assumed), cluster size, a coefficient of intracluster correlation (ICC or *k*), and an indication of its uncertainty	7/8
7b	When applicable, explanation of any interim analyses and stopping guidelines		N/A
**Randomisation**			
Sequence generation:			
8a	Method used to generate the random allocation sequence		4
8b	Type of randomisation; details of any restriction (such as blocking and block size)	Details of stratification or matching if used	4
Allocation concealment mechanism:			
9	Mechanism used to implement the random allocation sequence (such as sequentially numbered containers), describing any steps taken to conceal the sequence until interventions were assigned	Specification that allocation was based on clusters rather than individuals and whether allocation concealment (if any) was at the cluster level, the individual participant level, or both	N/A
Implementation:			
10	Who generated the random allocation sequence, who enrolled participants, and who assigned participants to interventions	Replaced by 10a, 10b, and 10c	N/A
10a		Who generated the random allocation sequence, who enrolled clusters, and who assigned clusters to interventions	4

Supplementary Table S5 (continued)

Section/topic and item No	Standard checklist item	Extension for cluster designs	Page No*
10b		Mechanism by which individual participants were included in clusters for the purposes of the trial (such as complete enumeration, random sampling)	4
10c		From whom consent was sought (representatives of the cluster, or individual cluster members, or both) and whether consent was sought before or after randomisation	4
Blinding:			
11a	If done, who was blinded after assignment to interventions (for example, participants, care providers, those assessing outcomes) and how		N/A
11b	If relevant, description of the similarity of interventions		N/A
Statistical methods:			
12a	Statistical methods used to compare groups for primary and secondary outcomes	How clustering was taken into account	8,9
12b	Methods for additional analyses, such as subgroup analyses and adjusted analyses		9
**Results**			
Participant flow (a diagram is strongly recommended):			
13a	For each group, the numbers of participants who were randomly assigned, received intended treatment, and were analysed for the primary outcome	For each group, the numbers of clusters that were randomly assigned, received intended treatment, and were analysed for the primary outcome	9
13b	For each group, losses and exclusions after randomisation, together with reasons	For each group, losses and exclusions for both clusters and individual cluster members	9
Recruitment:			
14a	Dates defining the periods of recruitment and follow-up		5
14b	Why the trial ended or was stopped		N/A
Baseline data:			
15	A table showing baseline demographic and clinical characteristics for each group	Baseline characteristics for the individual and cluster levels as applicable for each group	10
Numbers analysed:			
16	For each group, number of participants (denominator) included in each analysis and whether the analysis was by original assigned groups	For each group, number of clusters included in each analysis	10/11
Outcomes and estimation:			
17a	For each primary and secondary outcome, results for each group, and the estimated effect size and its precision (such as 95% confidence interval)	Results at the individual or cluster level as applicable and a coefficient of intracluster correlation (ICC or *k*) for each primary outcome	10/11
17b	For binary outcomes, presentation of both absolute and relative effect sizes is recommended		10/11
Ancillary analyses:			
18	Results of any other analyses performed, including subgroup analyses and adjusted analyses, distinguishing prespecified from exploratory		N/A
Harms:			
19	All important harms or unintended effects in each group (for specific guidance see CONSORT for harms106)		N/A
**Discussion**			
Limitations:			
20	Trial limitations, addressing sources of potential bias, imprecision, and, if relevant, multiplicity of analyses		13
Generalisability:			
21	Generalisability (external validity, applicability) of the trial findings	Generalisability to clusters and/or individual participants (as relevant)	13

Supplementary Table S5 (continued)

Section/topic and item No	Standard checklist item	Extension for cluster designs	Page No*
Interpretation:			
22	Interpretation consistent with results, balancing benefits and harms, and considering other relevant evidence		14
**Other information**			
Registration:			
23	Registration number and name of trial registry		4
Protocol:			
24	Where the full trial protocol can be accessed, if available		4
Funding:			
25	Sources of funding and other support (such as supply of drugs), role of funders		15

*Page numbers optional depending on journal requirements.
